# A multi-modal fusion scheme for the enhancement of PET/MR viewing

**DOI:** 10.1186/2197-7364-2-S1-A32

**Published:** 2015-05-18

**Authors:** Marco Aiello, Serena Monti, Marianna Inglese, Ernest Forte, Carlo Cavaliere, Onofrio Antonio Catalano, Emanuele Nicolai, Marco Salvatore

**Affiliations:** IRCCS SDN, Naples, Italy; Dipartimento di Elettronica, Informazione e Bioingegneria (DEIB), Politecnico di Milano, Italy; Università degli studi di Napoli, Parthenope, Italy

PET/MR imaging offers the possibility to achieve in one-shot both functional information provided by PET imaging and morpho-functional information with excellent soft tissue contrast provided by MRI. As a result of a typical PET/MR acquisition, each voxel carries a large amount of multivariate information that can be combined into a single image for a synthetic multi-parametric viewing (image fusion). This work is aimed to explore and introduce a reliable fusion scheme able to blend the useful information carried out from each modality into a single meaningful image. The proposed approach consists of a transformation of the source images followed by a blending and a consecutive reconstruction of fused image into the original domain. Following this scheme, three different transformations of source images (wavelet domain, Fourier domain and identity) and two blending procedures (alpha-blending and gamma-blending) were considered. For a comprehensive assessment of the fusion schemes under investigation, the results were evaluated both qualitatively and quantitatively on a dataset of 60 naturally coregistered. FDG-PET/MR studies of different anatomical districts in presence of neurological as well as oncological findings. The quality of fused images was assessed by experts who visually evaluated the loss of useful information with respect to the original modalities. Quantitatively, the information loss was estimated by means of a boundaries-preservation based metric as well as a metric based on mutual information. Preliminary results show that the fusion scheme composed by a wavelet-domain transform and a gamma-blending better depicts useful information of the original modalities. In particular, the proposed approach allows to better unveil the MR signal underlying PET signal in fused images in oncological as well as neurological studies. Furthermore, the use of a gamma-blending procedure ensures the best performances in a fully automated manner.

